# RREB1-mediated SUMOylation enhancement promotes chemoresistance partially by transcriptionally upregulating *UBC9* in colorectal cancer

**DOI:** 10.3389/fphar.2024.1381860

**Published:** 2024-07-23

**Authors:** Ya-nan Deng, Ying Chen, Shan Gao, Nan Zhang, Yinheng Luo, Shu Luo, Qiu Li, Xianghui Fu, Shufang Liang

**Affiliations:** ^1^ Department of Biotherapy, Cancer Center and State Key Laboratory of Biotherapy, West China Hospital, Sichuan University, Chengdu, China; ^2^ Department of Medical Oncology, Suining First People’s Hospital, Suining, Sichuan, China; ^3^ Department of Medical Oncology, Cancer Center, West China Hospital, Sichuan University, Chengdu, China

**Keywords:** SUMOylation, colorectal cancer, chemoresistance, 5-FU, RREB1, *UBC9*

## Abstract

Chemoresistance is a main cause of chemotherapy failure and tumor recurrence. The effects of global protein SUMOylation on chemoresistance in colorectal cancer (CRC) remains to be investigated. Herein, we have proposed that the elevated SUMO2/3-modified proteins confer 5-fluorouracil (5-FU) chemoresistance acquisition in CRC. The SUMOylation levels of global proteins in CRC cell lines were elevated compared with normal colon cell line NCM460. 5-FU treatment obviously reduced SUMOylation of global proteins in 5-FU-sensitive CRC cells including HT29, HCT116 and HCT-8. However, in 5-FU-resistant HCT-8/5-FU cells, the expression level of SUMO2/3-modified proteins was increased under 5-FU exposure in a concentration-dependent manner. 5-FU treatment combined with SUMOylation inhibitor ML-792 significantly increased the sensitivity of 5-FU-resistant cells to 5-FU and reduced colony formation numbers in HCT-8/5-FU cells. And UBC9-mediated SUMOylation elevation contributes to 5-FU resistance in HCT116 cells. Moreover, we also identified RREB1 as a regulator of SUMOylation profiling of global cellular proteins via directly binding to the promoter of *UBC9*. Overexpression of RREB1 promoted 5-FU resistance in CRC, which was partially abolished by treatment of inhibitor ML-792. In conclusion, RREB1-enhanced protein SUMOylation contributes to 5-FU resistance acquisition in CRC.

## 1 Introduction

Colorectal cancer (CRC) is a common malignant tumor of the digestive tract. It has large number of new cases every year and is gradually showing a trend of younger age, seriously endangering public health (Siegel et al., 2020). The treatment of colorectal cancer mainly includes surgical treatment, radiotherapy, chemotherapy, target therapy and the increasing immunotherapy in recent years. In China, the current treatment methods are still mainly surgical resection and chemotherapy. Due to the low early screening rate of CRC in China, most CRC patients are diagnosed in the middle and late stages with local or systemic metastasis.

So far, chemotherapy is still the main treatment for CRC. The commonly used chemotherapy regimens are mostly combination regimens based on 5-fluorouracil (5-FU), oxaliplatin and irinotecan (Sethy and Kundu, 2021). However, chemotherapy also has disadvantages such as poor efficacy and large side effects. Patients’ tolerance to chemotherapy drugs is also an important reason affecting efficacy. Some patients have no response to chemotherapy from the beginning, that is, innate chemotherapy tolerance. While others develop drug resistance during the treatment process, that is, acquired resistance. Therefore, exploring cell mechanism of chemotherapy drug resistance is of great significance to reverse drug resistance and improve patient chemotherapy efficacy.

Role of SUMOylation in chemotherapy has also been focused in recent years (Du et al., 2021; Zhang et al., 2022). Small ubiquitin-like modifier (SUMO) is a 11-kDa protein that is conjugated to the lysine amino acid (K) of proteins through sequential enzyme-catalyzed activation reactions, forming a specific protein post-translational modification (PTM), termed SUMOylation. The enzymes involved in the SUMOylation process mainly include SUMO-activating enzyme subunit 1 (SAE1), SUMO-activating enzyme subunit 2 (SAE2), SUMO-conjugating enzyme (UBC9) and SUMO E3 ligase (Yeh, 2009). SUMOylation is widely involved in regulation of cellular life activities, including cell cycle regulation, transcriptional regulation, protein sorting, localization, protein stability regulation, DNA damage response, cellular aging, as well as tumor cell proliferation and migration in a dynamic regulatory manner (Zhao, 2007). After the SUMO-modified substrate proteins have exerted their functions, the SUMO/sentrin specific protease (SENP) protein will remove the SUMO-modifier from the substrate protein.

Proteins often exert a specific function by many functionally similar proteins working together, and SUMO modification will regulate this class of proteins (Seeler and Dejean, 2017). For instance, UBC9 regulates a subset of KRAS-associated SUMOylated proteins (KASPs), including KAP1, CHD1 and EIF3L, which collectively support the anchorage-independent growth in KRAS-mutant CRC (Yu et al., 2015).

However, the overall change in protein SUMOylation modification in chemotherapy drugs for CRC has not been extensively investigated. In this study, we have explored the role of global protein SUMOylation profiling in response to chemotherapy drug exposure and drug resistance in CRC, and demonstrated that an elevation of global SUMO2/3-modified substrate proteins plays a crucial role in 5-FU resistance acquisition. Moreover, treatment by the SUMOylation inhibitor ML-792 partially reverses tolerance of drug-resistant cells to 5-FU. These new findings are promising to provide new options for reversing 5-FU tolerance in CRC.

## 2 Materials and methods

### 2.1 Cell culture

CRC cells HCT116, HT29, SW620, HCT-8 were collected in our laboratory, and a 5-FU-resistant cell line HCT-8/5-FU and its parental HCT-8 cell line were ordered from Hunan Fenghui Biotechnology Co., Ltd. HCT116, HT29 and SW620 cells were cultured in Dulbecco’s Modified Eagle Medium (DMEM). HCT-8 and HCT-8/5-FU cells were cultured in RPMI-1640 supplemented with 10% fetal bovine serum (FBS), 100 U/mL penicillin and 100 μg/mL streptomycin. The HCT-8/5-FU cells ordered from Hunan Fenghui Biotechnology Co., Ltd., possess a certain initial tolerance to 5-FU, with a resistance index (RI) of around 10. Subsequently, we further induced the cells with 5-FU to enhance the resistance to 5-FU, gradually increasing the concentration from 200 μM, 400 μM, 800 μM–1,600 μM over a period of approximately 5 months. The HCT-8/5-FU cells were passaged no more than 20 times, and 50 μM 5-FU was added to the culture medium on a daily basis to maintain their resistance to 5-FU.

### 2.2 Western blot

Cell lysates used for Western blot analysis was prepared as our published methods (Chen et al., 2021; Sheng et al., 2023). Specific primary antibodies: rabbit anti-SUMO1 (ET1606-53, HuaBio), rabbit anti-SUMO2/3 (ET1701-17, HuaBio), rabbit anti-UBC9 (ET1610-21, HuaBio), rabbit anti-SAE1 (ET7108-22, HuaBio), rabbit anti-SAE2 (ET1705-73, HuaBio), mouse anti-Flag (F1804, Sigma-Aldrich). Mouse anti-β-Tubulin (TA-10, Zsbio) and mouse anti-β-Actin (TA-09, Zsbio) were used for internal control. The primary antibodies were diluted at 1:1,000, and second antibodies were diluted at 1:10000. For global proteins SUMOylation detection, a final concentration of 20 mM N-ethylmaleimide (NEM) (HY-D0843, sigma) was added to RIPA lysis buffer.

### 2.3 Cell proliferation, IC_50_ detection, colony formation assay and EdU assay

Cell proliferation and IC_50_ detection was measured by cell counting kit-8 (CCK-8) assay referred as our previous publications (Yang et al., 2019). 2 × 10^3^ cells/well of HCT-8 or HCT-8/5-FU were seeded into a 96-well plate for indicated time. According to the manufacturer’s instruction, 10 µL CCK-8 reagent was added into 100 µL DMEM medium in each well for 2 h incubation at 37°C, then absorbance was measured at 450 nm. For IC_50_ detection, cells were seeded in 96-well plate and cultured with different concentration of drugs for 48 h. As for colony formation assay, 1,000 cells were seeded in 6-well plate with or without treatment of drugs, and cultured for 12–16 days.

EdU assay was performed in accordance with manufacturer’s instruction (KTA 2031, Abbkine). Briefly, EdU agent was supplied to cell culture with a final concentration at 50 µM for 6 h. After fixation, click-reaction and DAPI staining, images were taken with a fluorescence microscope.

### 2.4 Cell cycle

Flow cytometry was conducted using a Novoexpress system. For cell cycle assays, we first seeded 1 × 10^5^ cells in 6-well plate and FBS starvation was treated for 24 h. Then a cell cycle detection kit (KGA511, KeyGen BioTECH) was used to prepare the cells for cell cycle according to the manufacturer’s instructions.

### 2.5 Dual-fluorescence reporter assay

The promoter sequence of *UBC9* gene (Gene ID:7329) was cloned into firefly luciferase reporter plasmid pGL3-Basic (Chen et al., 2014) at *Mlu*Ⅰ and *Xho*Ⅰ sties (pGL3-*UBC9*), and the sequence was confirmed by DNA sequencing. The *Renilla* luciferase was used as an internal control. The Ras-responsive element binding protein 1 (RREB1) binding sequence in promoter of *UBC9* and *SAE1* was predicted in online website (https://jaspar.genereg.net/). Briefly, 2 kb sequence upstream of transcription start site was downloaded from NCBI in a FASTA-formatted style and was imported into JASPAR website to scan RREB1 binding motif (ID: MA0073.1). Relative profile score threshold was set at 80%.

pFlag-RREB1 and pFlag-RREB1^3KR^ plasmids were generated in our laboratory. In detail, RREB1 coding sequence (GI: 1519315172) was cloned into pCMV6-entry-Flag plasmid between *Sgf* Ⅰ and *Xho* Ⅰ (pFlag-RREB1). On the basis of pFlag-RREB1 plasmid, we mutated three lysine (K) amino acids into arginine (R) amino acids at sites 551, 885 and 913 to generate a pFlag-RREB1^3KR^ plasmid, expressing a mutant RREB1 protein with a lack of SUMOylation modification.

### 2.6 Statistical analysis

Statistical analysis was performed as required. Two-tailed unpaired Student’s t-test was applied to compare two groups of data. Two-way ANOVA was used to analyze the statistical of cell cycle distribution. Data was represented as mean ± SD (standard deviation). *p*-value <0.05 was considered as a statistically significant difference.

## 3 Results

### 3.1 5-FU significantly reduces SUMOylation of global proteins in CRC cells

**FIGURE 1:**
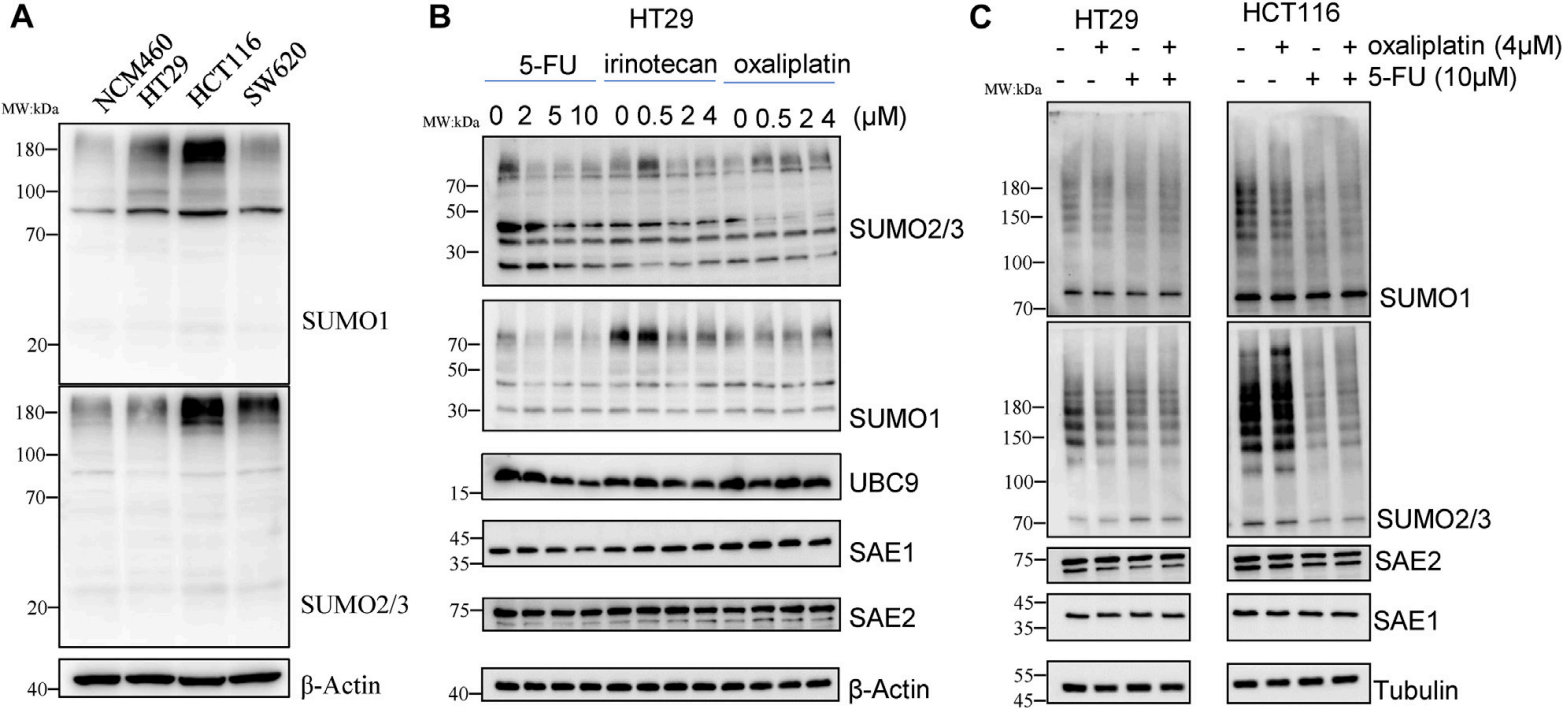
5-FU reduces SUMOylation level of the global proteins in CRC cells. **(A)** Global protein SUMOylation in NCM460, HT29, HCT116 and SW620 cells. **(B)** Cells were harvested for Western blot analysis after treatment by 5-FU, irinotecan or oxaliplatin at indicated concentrations for 24 h. **(C)** Global protein SUMOylation profiling was confirmed in HT29 and HCT116 cells with treatment of 10 µM 5-FU or 4 µM oxaliplatin.

We first detected SUMOylation level of global proteins under chemotherapy drug exposure in CRC cell lines, including HCT116, HT29 and SW620 and normal colon epithelial NCM460 cells. As results, levels of SUMO1 and SUMO2/3 modifications of global proteins in CRC cell lines were significantly higher than those in normal colon epithelial cells NCM460 (Figure 1A), suggesting that SUMOylation modification is linked with occurrence and development of CRC.

To investigate whether SUMOylation modification is involved in the response of chemotherapy drug treatment, HT29 cells were treated with different concentrations of 5-FU, irinotecan and oxaliplatin for 24 h, and then changes of global protein SUMOylation profiling were detected by Western blot. The results showed that irinotecan and oxaliplatin had little effect on the SUMOylation level of global proteins, while 5-FU obviously reduced the SUMOylation modification of total proteins in a concentration-dependent manner. The downregulation of global protein modified by SUMO2/3 was more obvious than that modified by SUMO1. 10 μM 5-FU treatment downregulated the level of SUMO2/3-modified global proteins to 0.45-fold compared with no 5-FU exposure. We also detected the enzymes involved in SUMOylation process, among which the expression level of SAE2 did not show obvious changes under chemotherapy drug exposure, while the expression of SAE1 and UBC9 level was respectively decreased to 0.45-fold and 0.38-fold under 10 µM 5-FU treatment (Figure 1B), suggesting that global proteins SUMOylation may play a role in 5-FU response.

We further treated HCT116 and HT29 cells with 4 µM oxaliplatin or 10 µM 5-FU to validate the effect of chemotherapy agents on the decrease of SUMOylation in CRC. The results showed that 5-FU treatment reduced the level of SUMO2/3-modified global proteins in HCT116, while oxaliplatin treatment cause a little downregulation of the level of SUMO2/3-modified global proteins (Figure 1C), demonstrating that the SUMOylation modification of global proteins is sensitive to 5-FU exposure, but not to irinotecan and oxaliplatin. These results suggest that global proteins SUMOylation modification, especially SUMO2/3-modified global proteins, may play an important role in 5-FU therapy in CRC cells.

### 3.2 Establishment and characteristics analysis of 5-FU-resistant HCT-8/5FU cell line

**FIGURE 2:**
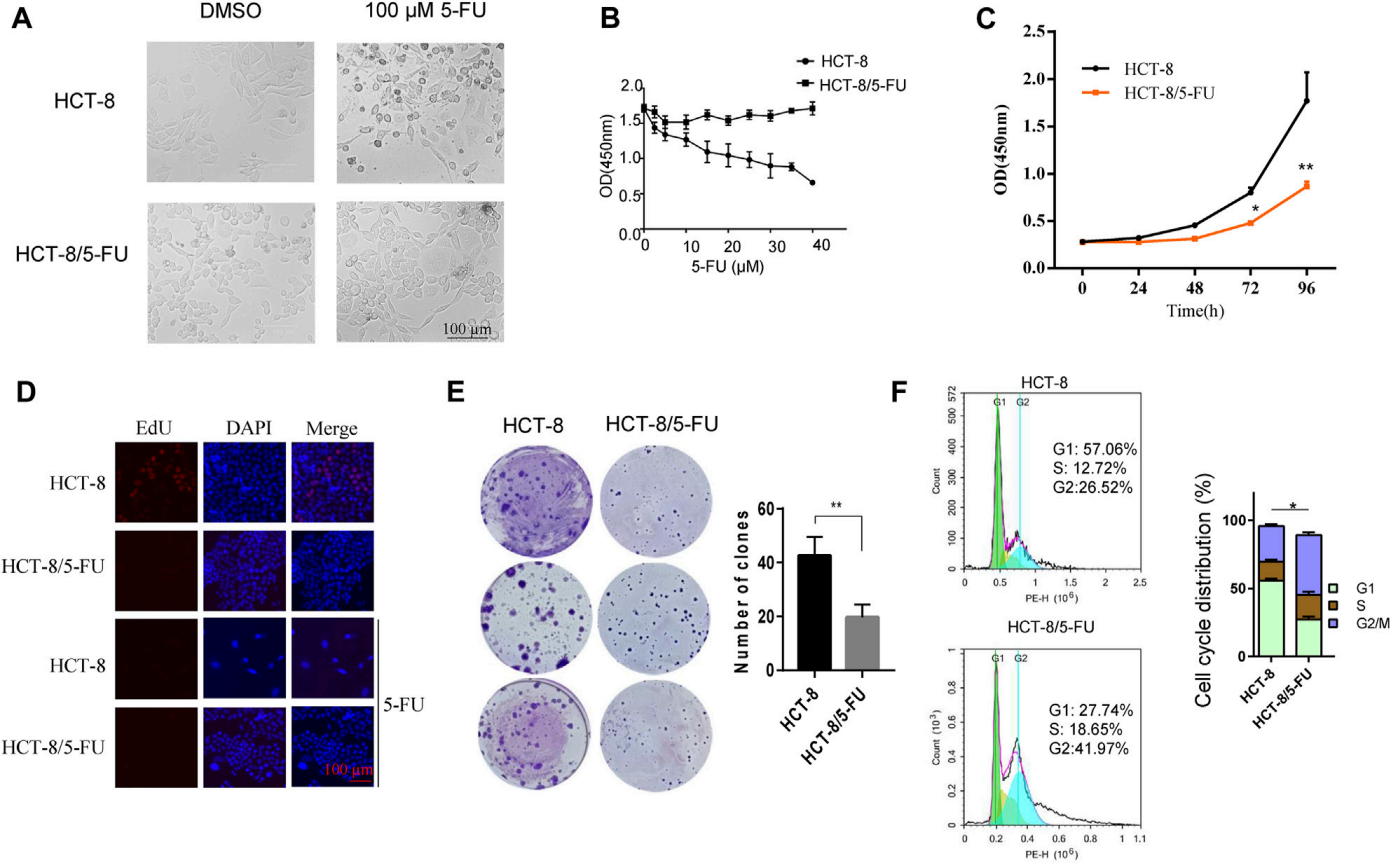
5-FU-resistant HCT-8/5FU cell line establishment and characteristics analysis. **(A)** Morphology observation of HCT-8 and HCT-8/5-FU after treatment of 100 µM 5-FU or DMSO for control after 24 h. **(B)** Cell viability measurement of HCT-8 cells and HCT-8/5-FU cells after treatment of 5-FU with concentrations from 0 μM to 40 µM for 48 h. **(C)** Cell proliferation was measured by CCK-8 assay at 0, 24, 48, 72, 96 h in HCT-8 cells and HCT-8/5-FU cells. **(D)** EdU assay was performed in HCT-8 cells and HCT-8/5-FU cells with or without a 5-FU treatment. Scale bar:100 µm. **(E)** 500 cells/well of HCT-8 or HCT-8/5-FU were seeded in 6-well plate for colony formation assay. After 16 days, cells were fixed and stained with crystal violet. **(F)** Cell cycle analysis was performed in HCT-8 and HCT-8/5-FU. *p < 0.05, **p < 0.01.

A 5-FU-resistant cell line HCT-8/5-FU was established by inducing partial 5-FU-tolerant HCT-8/5-FU cells continuously with 5-FU induction. Compared with the parental HCT-8 cells, HCT-8/5-FU cells showed a significant increase in tolerance to 5-FU, resistance index (RI) is 56. After treatment with 20 µM 5-FU, parental HCT-8 cells showed obvious apoptotic morphology such as cell shrinkage and floating, while the morphology of 5-FU-resistant HCT-8/5-FU cells did not change significantly and still maintained adherent growth (Figure 2A). We further compared cell response characterization under 0–40 µM 5-FU exposure between HCT-8 cells and HCT-8/5-FU cells, and found that 5-FU treatment gradually decreased cell viability of HCT-8 cells, with the OD value (measured at 450 nm) dropping from 1.7 to 0.6. However, 40 µM 5-FU exposure had a little influence on cell viability of HCT-8/5-FU cells, indicating that our established HCT-8/5-FU cells harbored a 5-FU-resistance ability (Figure 2B).

Next, we measured the growth characteristics of HCT-8 and 5-FU-resistant HCT-8/5-FU cells by CCK-8, EdU and colony formation assay. HCT-8/5-FU cells showed weaker cell viability compared with HCT-8 cells from 48 to 96 h. At 96 h, the OD (measured at 450 nm) of HCT-8 reached 1.770 ± 0.301, while the OD of HCT-8/5-FU was significantly lower (0.867 ± 0.053, *p* < 0.01) (Figure 2C). We next performed an EdU assay in HCT-8 and HCT-8/5-FU cells to monitor the cell proliferation. The results showed that the EdU-positive cells in HCT-8 cells reached 20% (32/160), which is obviously higher than that in HCT-8/5-FU cells (almost none EdU-positive cells in HCT-8/5-FU). In addition, 5-FU treatment induces the cell apoptosis in HCT-8 cells, thereby dramatically reducing the proportion of EdU-positive cells in HCT-8 (Figure 2D).

The colony formation number of HCT-8/5-FU cells was significantly lower than that of HCT-8 cells (19.6 ± 4.72 vs. 42.67 ± 6.8, *p* < 0.01) (Figure 2E). These results demonstrated that HCT-8/5-FU cell line decreased its cell proliferation and colony formation ability. Furthermore, flow cytometry analysis revealed that the cell cycle of HCT-8/5-FU was reset, with a significant increased proportion in G2/M phase (*p* = 0.02) (Figure 2F). Together, our results showed that HCT-8/5-FU slows down cell proliferation rate by reprogramming cell cycle, thereby increasing tolerance to 5-FU.

### 3.3 SUMO2/3-modified proteins were specially elevated in 5-FU-resistant HCT-8/5-FU cells under 5-FU treatment

**FIGURE 3:**
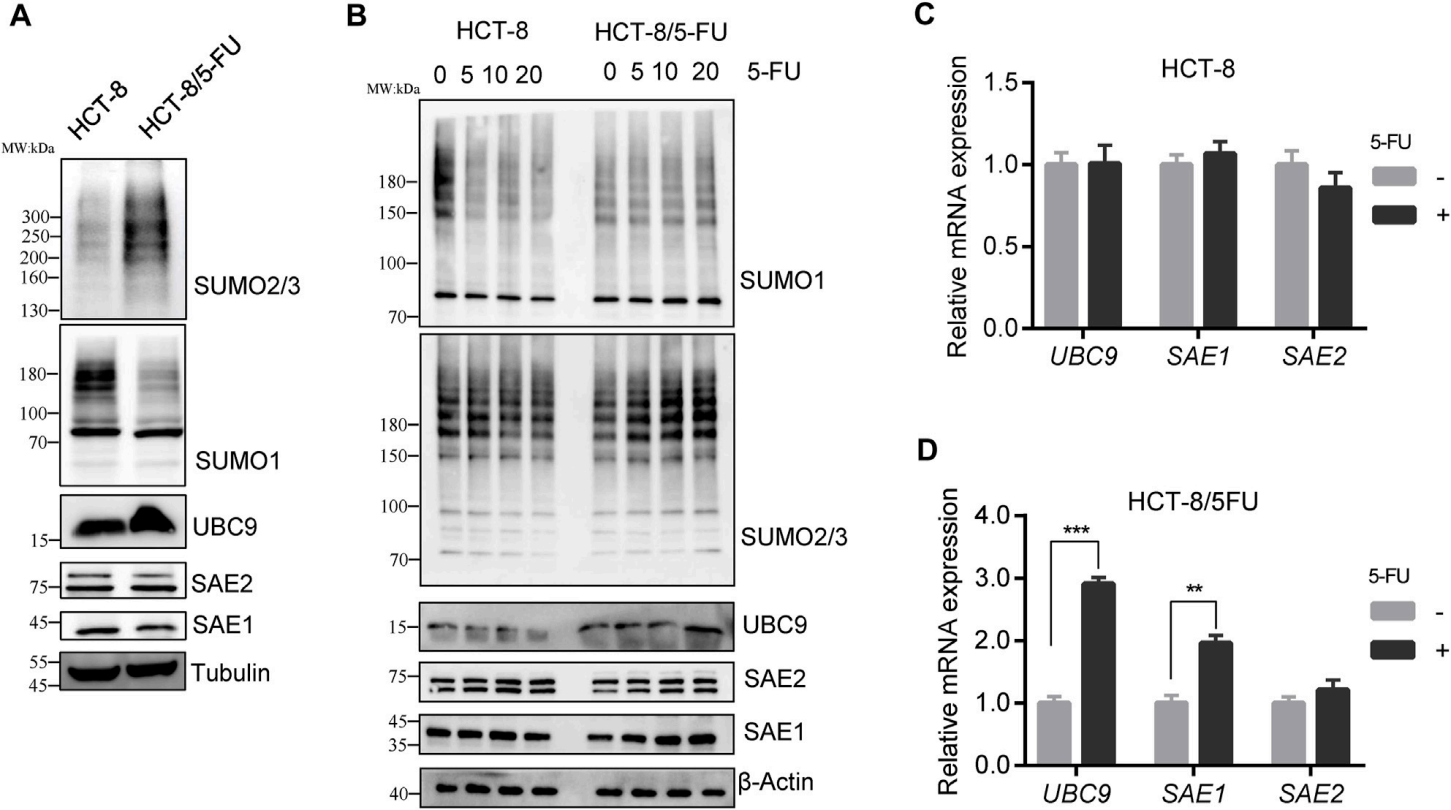
SUMO2/3 modified proteins were specially elevated in 5-FU resistant HCT-8/5-FU cells under 5-FU treatment. **(A)** HCT-8/5-FU cells were cultured without 5-FU exposure for 1 week, then cell lysates of HCT-8/5-FU and parental HCT-8 cells were prepared for the global protein SUMOylation detection. **(B)** HCT-8/5-FU cells and parental HCT-8 cells that have been removed of 5-FU for a week were retreated with 5-FU for 24 h, then cells were harvested for Western blot analysis. **(C,D)** mRNA expression of UBC9, SAE1 and SAE2 were detected in HCT-8 cells or HCT-8/5-FU cells with or without 100 µM 5-FU treatment for 48 h by qPCR. **p < 0.01, ***p < 0.001.

We investigated the total SUMOylation modification profiling of cell global proteins in HCT-8/5-FU and HCT-8 cells. To exclude cell culture condition difference between HCT-8 and HCT-8/5-FU, HCT-8/5-FU cells were cultured in RPMI-1640 for 1 week without 5-FU supplement, proteins were collected for Western blot analysis. The level of whole SUMO1-modified proteins in HCT-8/5-FU was lower than that in parental HCT-8 cells, while the level of SUMO2/3-modified proteins was elevated in HCT-8/5-FU (Figure 3A). The decrease in the level of SUMO1-modified proteins was consistent with the expression downregulation of total proteins caused by 5-FU exposure in HCT116 and HT29 cells, indicating that the level of some SUMO1- modified proteins in HCT-8/5-FU continued to decrease during continuous induction of 5-FU. In contrast, the level of global SUMO2/3-modified proteins was upregulated under continuous 5-FU induction in HCT-8/5-FU cells.

Similarly, HCT-8/5-FU and HCT-8 cells were re-treated with different concentrations of 5-FU for 24 h to validate the SUMOylation level. In parental HCT-8 cells, 20 µM 5-FU treatment downregulated the level of global SUMO1-modified proteins and SUMO2/3-modified proteins. However, re-treatment with 5-FU in HCT-8/5-FU did not downregulate the level of SUMO1-modified proteins, but instead, it increased the level of SUMO2/3-modified proteins in a concentration-dependent manner. Moreover, the protein expression levels of UBC9 and SAE1 in HCT-8/5-FU were gradually increased in a concentration-dependent manner under 5-FU treatment.

On the contrary, 20 µM 5-FU treatment in HCT-8 cells reduced the expression of UBC9 and SAE1 (Figure 3B). In addition, we measured the mRNA expression of *UBC9*, *SAE1* and *SAE2* in HCT-8 and HCT-8/5-FU cells with or without 5-FU exposure. In HCT-8 cells, mRNA expression of *UBC9*, *SAE1* and *SAE2* showed no changes under 5-FU treatment (Figure 3C). While, in 5-FU-resistant HCT-8/5-FU cells, 5-FU treatment significantly improved the expression of *UBC9* with 3-fold increase and expression of *SAE1* with 2-fold enhancement, but showed little influence on the mRNA expression of *SAE2* (Figure 3D). Our results indicated that increase of the SUMO2/3-modified proteins under 5-FU treatment is helpful for acquisition of 5-FU resistance in HCT-8/5-FU cells.

### 3.4 SUMOylation inhibition sensitizes HCT-8/5-FU cells to 5-FU

**FIGURE 4:**
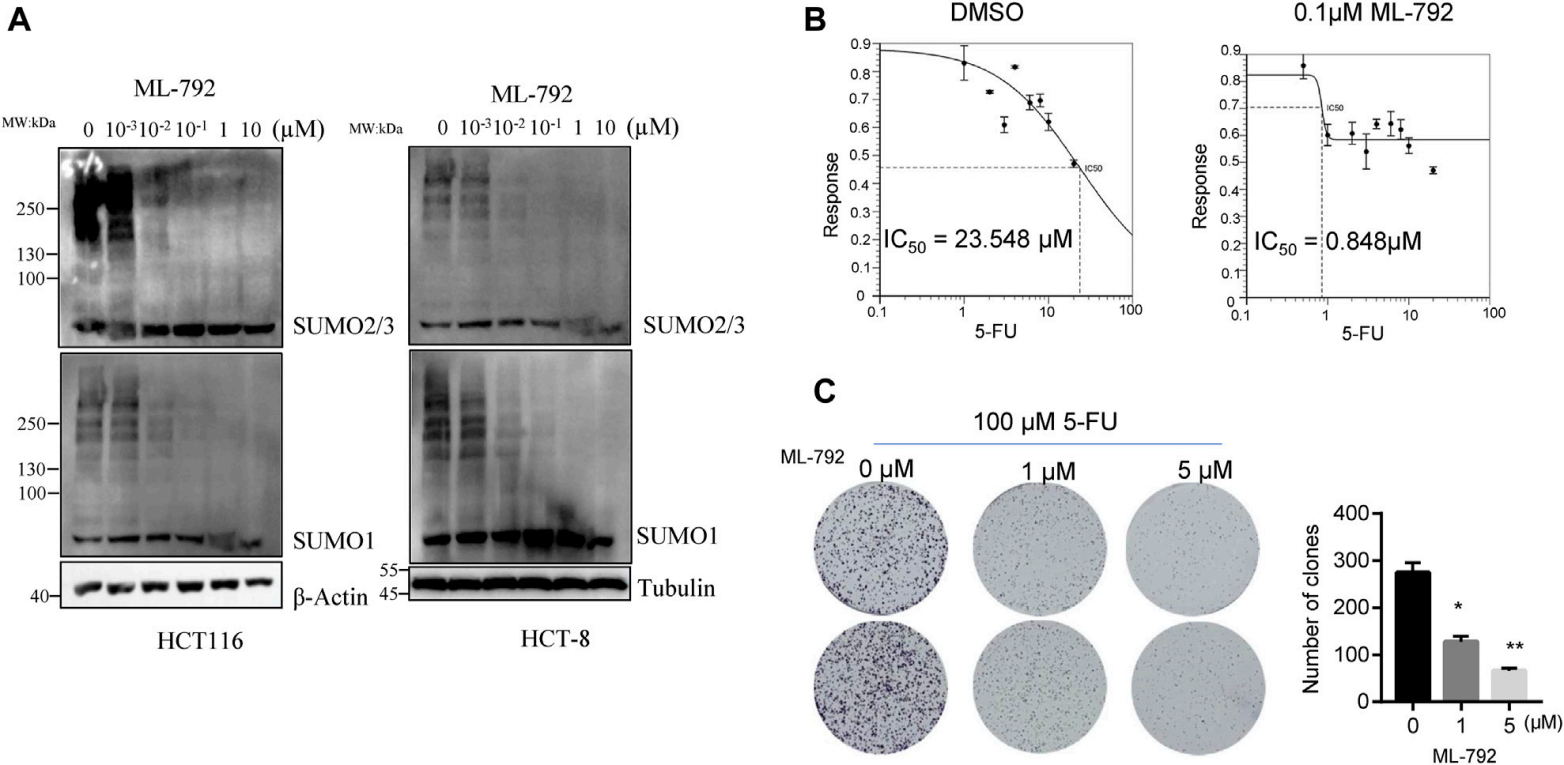
SUMOylation inhibitor, ML-792, reverses the 5-FU resistance in HCT-8/5-FU cell line. **(A)** SUMOylation inhibitor ML-792 inhibits the conjunction of SUMO1 and SUMO2/3 to substrates. **(B)** 5-FU IC_50_ detection was conducted in HCT-8 cells with incubation of 0.1 µM ML-792 or DMSO for control. **(C)** 1,000 cells/well of HCT-8/5-FU were seeded in 6-well plate for colony formation assay with combination incubation of 100 µM 5-FU and 0, one or 5 µM ML-792. *p < 0.05, **p < 0.01.

To uncover the role of SUMOylation in 5-FU resistance, we applied an inhibitor of SUMOylation, ML-792, to inhibit the global proteins SUMOylation modification and to observe the changes of cell tolerance to 5-FU. Firstly, we confirmed the inhibitory effect of ML-792 on CRC cells, and found that 0.1 µM ML-792 began to inhibit the conjunction of SUMO1 and SUMO2/3 on substrates (Figure 4A). 0.1 µM ML-792 greatly downregulated the IC_50_ of 5-FU in HCT-8 cells (IC_50_ = 0.848 µM) (Figure 4B). In addition, we applied a combination of 100 µM 5-FU with 0 μM, 1 µM or 5 µM ML-792 to compare colony formation influence of HCT-8/5-FU cells. ML-792 in combination with 5-FU treatment significantly reduced survival ability of HCT-8/5-FU cells (Figure 4C). These results confirmed that SUMOylation plays an important positive role in 5-FU resistance and that inhibition of global proteins SUMOylation can reverse 5-FU resistance.

### 3.5 Overexpression of UBC9 and SAE1 promotes the 5-FU resistance in CRC

**FIGURE 5:**
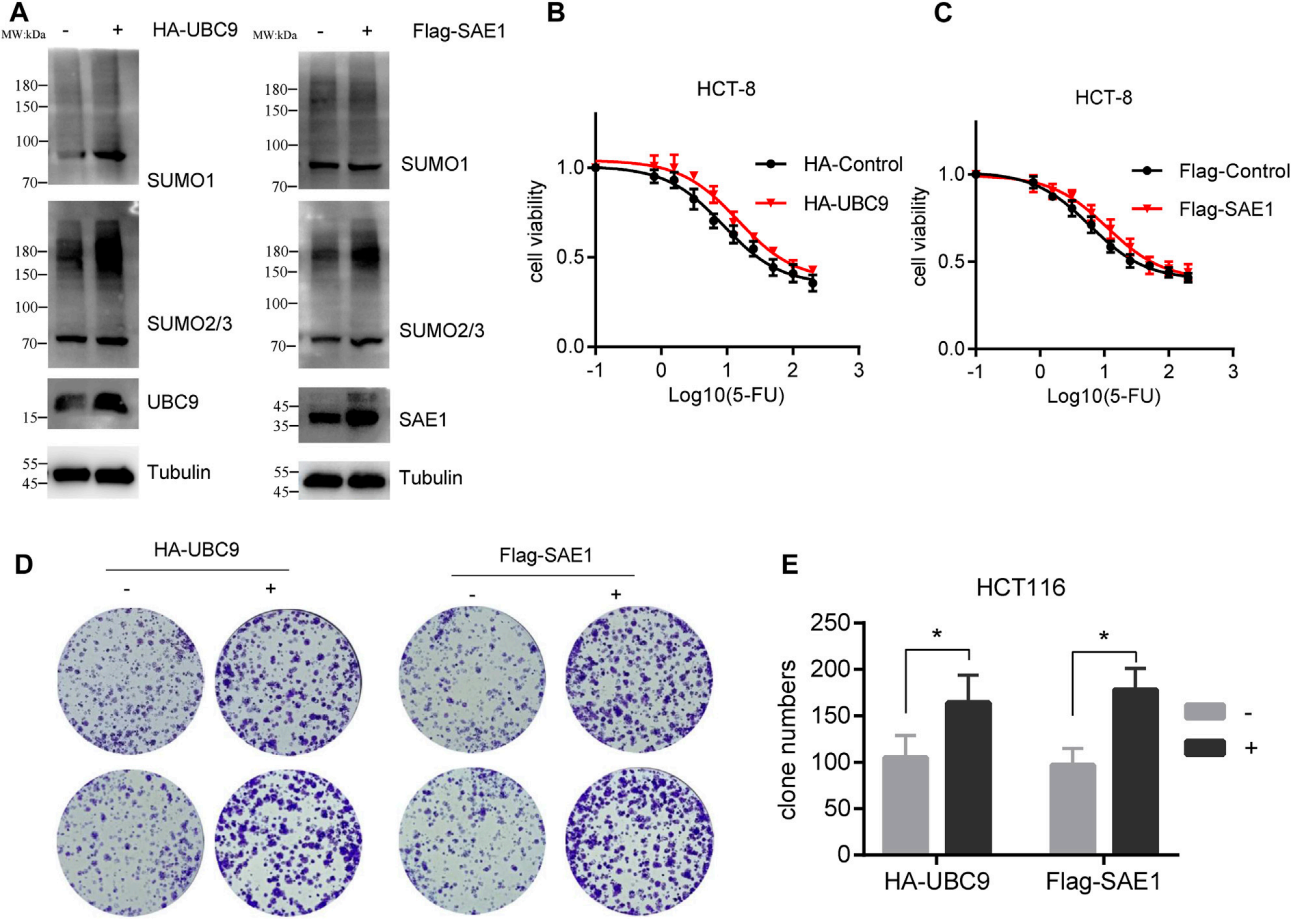
Overexpression of UBC9 or SAE1 enhances the 5-FU resistance in HCT116 cells. **(A)** Overexpression of HA-UBC9 or Flag-SAE1 in HCT116 cells increased more SUMO2/3 modified protein levels than that of SUMO1. **(B,C)** 5-FU IC_50_ detection in HCT-8 cells with UBC9 overexpression or SAE1 overexpression. *Y*-axis was shown as percentage of control group (without 5-FU treatment). **(D)** Colony formation assay to examine the survival of HCT116 cells under 0.5 µM 5-FU treatment with or without overexpression of UBC9 or SAE1. **(E)** The clone survival numbers in colony formation assay performed in Figure **(D)** *p < 0.05.

Upon with 5-FU exposure, the expression level of UBC9 and SAE1 in HCT-8/5-FU cells has been enhanced (as shown in Figure 3), and we further investigate the potential role of these proteins in the acquisition of resistance to 5-FU in CRC. We transfected pHA-UBC9 or pFlag-SAE1 in HCT116 cells to increase the global proteins SUMOylation and found that overexpression of UBC9 and SAE1 elevated more SUMO2/3-modified proteins than SUMO1-modified proteins (Figure 5A). Overexpression of HA-UBC9 increased the IC_50_ of 5-FU from 8.7 µM to 14.5 µM in HCT-8 cells (Figure 5B). Moreover, the overexpression of Flag-SAE1 in HCT-8 also improved the cell viability (IC_50_ = 11.02 µM) under 5-FU treatment compared with Flag-Control group (IC_50_ = 5.9 µM) (Figure 5C). Next, we measured the effect of UBC9 and SAE1 on colony formation under 5-FU treatment in HCT116 cells. The results showed that overexpression of UBC9 promoted the survival number of HCT116 cells under 0.5 µM 5-FU treatment (164.2 ± 30.5 vs. 105.3 ± 24.4, *p* < 0.05). Similarly, overexpression of SAE1 also promoted the colony number of HCT116 cells (178.3 ± 23.5 vs. 97.4 ± 18.6, *p* < 0.05) (Figures 5D, E). Together, these results demonstrate that UBC9 and SAE1 are crucial for 5-FU resistance acquisition in CRC.

### 3.6 RREB1-mediated SUMOylation increase contributes to 5-FU resistance by upregulating UBC9

**FIGURE 6:**
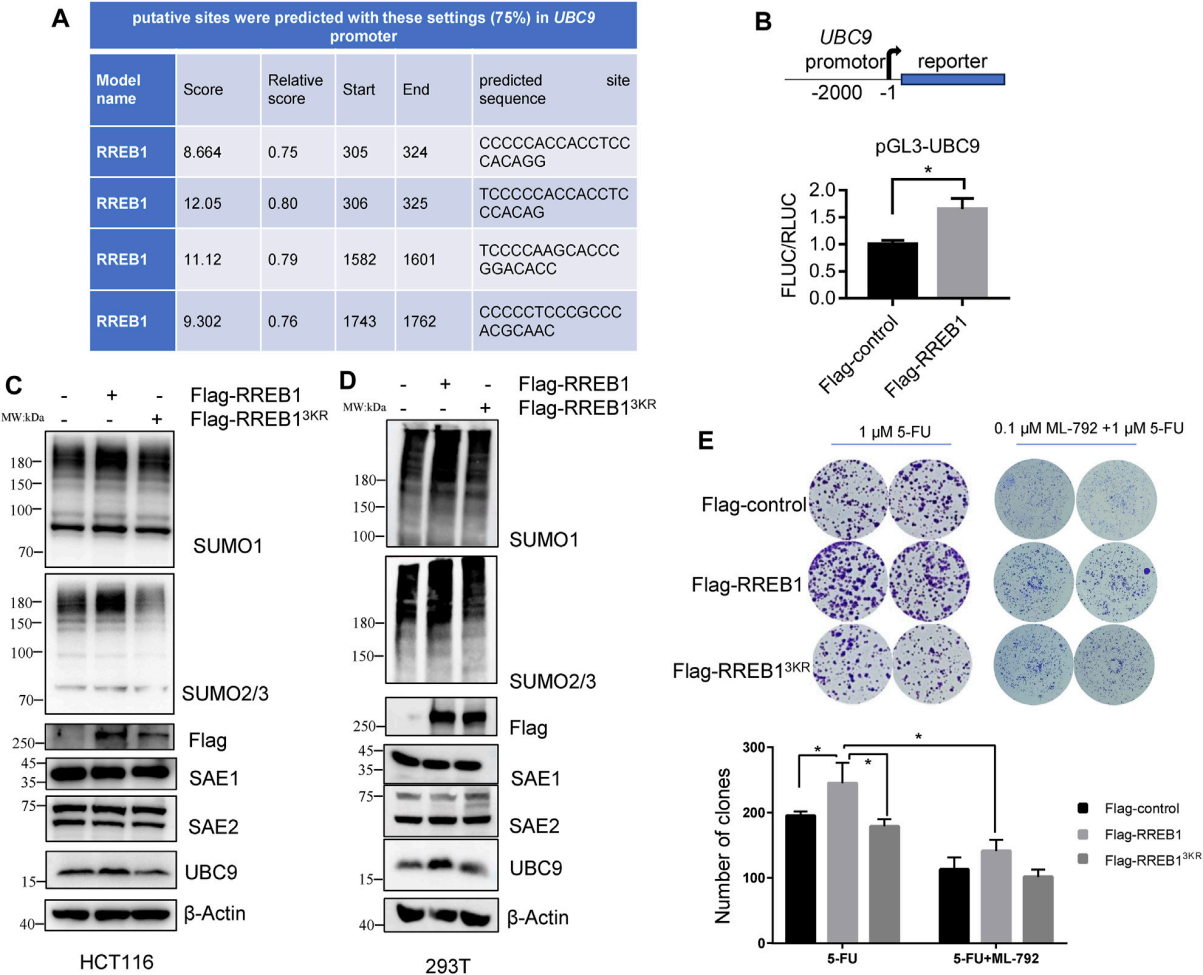
RREB1 promotes global proteins SUMOylation and contributes to 5-FU resistance by transcriptionally upregulating *UBC9* in CRC. **(A)** Putative binding sites of RREB1 in *UBC9* promoter were predicted in online website (https://jaspar.genereg.net/). Relative score >0.75 was considered as a binding site. **(B)** We cloned the *UBC9* promoter sequence (from the transcription start site to upstream 2000 base pair) into pGL3-basic plasmid to generate pGL3-*UBC9* plasmid. The expression of reporter gene was performed in HEK293T cells. **(C,D)** Transient transfection of pFlag-RREB1 or pFlag-RREB1^3KR^ into HCT116 or HEK293T cells for 48 h, cell lysates were prepared for Western blot analysis. **(E)** 1,000 cells/well of HCT116 cells that overexpressing RREB1 or RREB1^3KR^ were seeded in 6-well plate with incubation of 1 µM 5-FU independently or combination with 0.1 µM ML-792 for 14 days. *p < 0.05.

Analysis of the *UBC9* and *SAE1* promoter sequence revealed that *UBC9*, but not *SAE1* (data not shown), contained a motif recognized by RREB1 (Figure 6A), suggesting that RREB1 may directly bind to the *UBC9* promoter sequence. RREB1 is overexpressed in CRC and has been identified as a transcription factor to regulate the expression of multiple targets in CRC, including miR-143/145 (Kent et al., 2013) and ITGA7 (Li et al., 2018). The *UBC9* promoter sequence was cloned into a dual luciferase reporter gene vector to construct the pGL3-*UBC9* vector. pFlag-RREB1 plasmid or control plasmid pFlag-control together with pGL3-*UBC9* were co-transfected into HEK293T cells to detect the expression of the reporter gene. The results showed that RREB1 promoted the expression of UBC9 (Figure 6B).

Our team previously reported the oncogenic role of RREB1 in CRC development and migration (Deng et al., 2020; Chen et al., 2021). pFlag-RREB1 plasmid and mutated SUMOylation sites plasmid pFlag-RREB1^3KR^ were transiently transfected into HCT116 and HEK293T cells. Our results showed that RREB1 increase the expression level of SUMO1-modifed proteins and SUMO2/3-modified proteins, while the pFlag-RREB1^3KR^ plasmid with mutated SUMOylation sites abolished this effect. RREB1 also promoted the expression of the enzyme UBC9 but showed little influence on SAE1 expression (Figures 6C, D), which is consistent with the result of promoter prediction in *UBC9* and *SAE1*. We have now discovered that RREB1 also regulates the level of global protein SUMOylation.

In colony formation assay, we found that RREB1 but not RREB1^3KR^ significantly increased the number of clones of HCT116 under 1 µM 5-FU treatment, indicating that RREB1 could increase tolerance to 5-FU. In the presence of ML-792, RREB1 did not significantly increase the number of clones of HCT116 under 1 µM 5-FU treatment (Figure 6E). Our results demonstrate that the elevation of global protein SUMOylation modification mediated by RREB1 promotes the tolerance of CRC cells to 5-FU.

## 4 Discussion

SUMOylation is an important post-translational modification of proteins that plays a significant role in cell regulation, tumor occurrence and development, DNA damage response, and the development of pathogenic diseases (Celen and Sahin, 2020). Our research has shown that SUMOylation plays a crucial role in the tolerance of CRC to chemotherapy drugs, particularly 5-FU. Global protein SUMOylation was found to be increased in CRC cells.

In our studies, we have found that the change in SUMO2/3 modification of global proteins in CRC cells is more significant than SUMO1 modification after 5-FU treatment. In addition, the upregulation of SUMO2/3-modified proteins in HCT-8/5-FU cells suggests that SUMO2/3 plays a more important role than SUMO1 in 5-FU. A previous report (Bursomanno et al., 2015) also found that the DNA polymerase inhibitor aphidicolin (APH) could specifically induce the increase of SUMO2/3-modified proteins, while there was no significant change in SUMO1-modified proteins, indicating that SUMO1 and SUMO2/3 played different roles in replication stress. SUMO2/3-modified proteins are widely involved in the DNA damage repair process (Becker et al., 2013). In DNA damage induced by the DNA damaging agent methyl methanesulfonate (MMS), a study (Hendriks et al., 2015) identified 55 SUMO2-modified proteins involved in DNA damage repair, among which 20 proteins were upregulated and 33 proteins were downregulated.

Under chemotherapy drug treatment, the level of SUMO1 modification of some proteins decreases or the degradation of some SUMOylated proteins will show a decrease in global SUMOylation. This subset of proteins may play a more stressful role in chemotherapy drug treatment. After treatment with 5-FU in sensitive cells, this subset of proteins either undergoes protein level degradation or experiences a decrease in SUMO1 modification level. However, this subset of proteins may not play a significant role in acquiring tolerance to 5-FU. Importantly, we observed that the level of SUMO2/3 modification of this subset of proteins does not decrease after long-term 5-FU treatment in HCT-8/5-FU cells, and the proportion of proteins with increased SUMO2/3 modification with 5-FU treatment in global proteins is increasing. This may be an important reason for sensitive cells to acquire resistance to 5-FU. Although we have not yet identified the specific 5-FU-resistant proteins modified by SUMO2/3, we have confirmed the important role of SUMO2/3 modification in acquiring resistance to 5-FU by inhibiting the increase of global protein SUMOylation through the use of SUMOylation inhibitors. In future works, identifying these specific 5-FU-resistant proteins modified by SUMO2/3 and specifically inhibiting this subset of proteins will be important for reversing 5-FU resistance. Currently, research on proteins modified by SUMO2/3 related to 5-FU resistance is mostly scattered and individual, and comprehensive research is relatively rare. For example, loss of HDAC2 SUMOylation increases CRC sensitivity to 5-FU (Kiweler and Nicole, 2019). Elevated FOXK2 SUMOylation causes resistance to 5-FU in hepatocellular carcinoma (Li et al., 2023).

The enzymes involved in the SUMOylation machinery have a significant impact on global SUMOylation. We observed that the expression of SAE1 and UBC9 decreased under 5-FU treatment, while the expression of SAE2 did not change significantly. Moreover, in HCT-8/5-FU cells, UBC9 and SAE1 expression increased with increasing concentration of 5-FU, indicating that UBC9 and SAE1 may be an important target for reducing global SUMOylation modification by 5-FU. RREB1 elevates the level of global proteins SUMOylation by directly binding to the *UBC9* promoter sequence to increase UBC9 protein expression. This indicates that the most important factor for exerting 5-FU resistance function is still the final SUMOylated target protein rather than enzymes involved in the SUMO process. The phenomenon that RREB1 regulates enzymes involved in the SUMO process has been reported earlier; some teams found that RREB1 can regulate UBA2/SAE2 protein expression to promote CRC proliferation (Liu et al., 2022). Herein, we have proposed that *UBC9* is also a target gene for RREB1. Interestingly, our unpublished data have supported that the RREB1 itself is also a substrate for SUMOylation. In this study, the mutant deSUMOylated RREB1 has a weakened function on the expression of UBC9. A previous report (Kuppuswamy et al., 2008) shows that CtBP1 complex provides a platform for the SUMOylation of ZEB1, in which UBC9 and RREB1 are also components. However, whether the CtBP1 complex plays a role in the RREB1 SUMOylation process needs a further explore in the future.

Currently, there are numerous studies targeting SUMOylation, generally focusing on key enzymes involved in the SUMOylation process, such as SAE1, UBC9, and SENP1. Inhibitors targeting SAE1 include ML-93 (Biederstädt et al., 2020), ML-792 (He et al., 2017) and TAK-981 (Langston et al., 2021). Additionally, natural products like compound 9 and compound 21 have been identified as SAE1 inhibitors (Kumar et al., 2013a; Kumar et al., 2013b). Synthetic inhibitors targeting UBC9 include GSK145A (Brandt et al., 2013), compound 2 (Zlotkowski et al., 2017) and 2-D08 (Kim et al., 2013). Synthetic inhibitors targeting SENP1, such as compound 38 and compound J5, have also been developed. However, most of these inhibitors have not undergone clinical trials. TAK-981, a SUMOylation inhibitor currently in clinical trials, covalently binds to SAE1. Its therapeutic effects *in vivo* demonstrate significant inhibition of colorectal cancer tumor growth, upregulation of type I interferon in immune cells, and activation of interferon-dependent macrophages, T cells, natural killer cells, and dendritic cells (Khattar et al., 2019). TAK-981 is currently undergoing clinical trials as a monotherapy or in combination with anti-CD38 monoclonal antibodies, pembrolizumab, or anti-CD20 rituximab to assess its efficacy and safety.

Nevertheless, considering the crucial role of SUMOylation in normal biological activities such as cell cycle regulation, protein stability modulation and enzyme activity modulation, directly targeting global SUMOylation modification in disease therapy may lead to dysfunction and significant side effects. Therefore, targeting SUMOylated substrate proteins with drugs represents an effective alternative.

## Data Availability

The original contributions presented in the study are included in the article/Supplementary Material, further inquiries can be directed to the corresponding author.
